# The Effect of Roll Circular Vection on Roll Tilt Postural Responses and Roll Subjective Postural Horizontal of Healthy Normal Subjects

**DOI:** 10.3390/brainsci13111502

**Published:** 2023-10-24

**Authors:** Taylor W. Cleworth, John H. J. Allum, Emma I. Nielsen, Mark G. Carpenter

**Affiliations:** 1 School of Kinesiology and Health Science, York University, Toronto, ON M3J 1P3, Canada; tclewort@yorku.ca; 2 Centre for Vision Research, York University, Toronto, ON M3J 1P3, Canada; 3 Department of Otorhinolaryngology, University of Basel Hospital, CH-4031 Basel, Switzerland; 4 School of Kinesiology, University of British Columbia, Vancouver, BC V6T 1Z4, Canada; emma.nielsen@ubc.ca (E.I.N.); mark.carpenter@ubc.ca (M.G.C.)

**Keywords:** subjective postural horizontal, postural responses, circular vection, visual–vestibular interactions

## Abstract

*Background*: Falls and related injuries are critical issues in several disease states, as well as aging, especially when interactions between vestibular and visual sensory inputs are involved. Slow support surface tilt (0.6 deg/s) followed by subjective postural horizontal (SPH) assessments have been proposed as a viable method for assessing otolith contributions to balance control. Previous assessments of perceived body alignment to vertical, including subjective visual vertical, have suggested that visual inputs are weighted more when vestibular information is near the threshold and less reliable during slow body tilt. To date, no studies have examined the influence of visual stimuli on slow roll-tilt postural responses and the SPH. Therefore, this study investigated how dynamic visual cues, in the form of circular vection (CV), influence postural responses and the perception of the horizontal during and after support surface tilt. *Methods*: Ten healthy young adults (6 female, mean age 23) wore a head-mounted display while standing on a tilting platform. Participants were asked to remain upright for 30 s, during which (1) the visual scene rotated, inducing roll CV clockwise (CW) or counter-clockwise (CCW) at 60°/s; (2) the platform only (PO) rotated in roll to test SPH (0.6°/s, 2°, CW or CCW); (3) a combination of both; or (4) neither occurred. During SPH trials, participants used a hand-held device to reset the position of the platform to 0.8°/s to their perceived SPH. The angular motion of body segments was measured using pairs of light-emitting diodes mounted on the head, trunk and pelvis. Segment motion, prior to platform motion, was compared to that at peak body motion induced by platform motion and when SPH had been set. *Results*: When the support surface was tilted 2°, peak upper body tilt significantly increased for congruent CV and platform tilt and decreased at the pelvis for incongruent CV when compared to PO, leading to significant differences across body segments for congruent and incongruent conditions (*p* ≤ 0.008). During PO, participants’ mean SPH deviated from horizontal by 0.2°. The pelvis deviated 0.2°, the trunk 0.3°, and the head 0.5° in the direction of initial platform rotation. When platform tilt and CV directions were congruent or incongruent, only head tilt at SPH reset under congruent conditions was significantly different from the PO condition (1.7° vs. 0.5°). *Conclusions*: Roll CV has a significant effect on phasic body responses and a less significant effect on tonic body responses to lateral tilt. The SPH of the support surface was not altered by CV. Responses during tilt demonstrated enhanced reactions for congruent and reduced reactions for incongruent CV, both different from responses to CV alone. Tonic body displacements associated with SPH were changed less than those during tilt and were only slightly larger than displacements for CV alone. This study supports the hypothesis of weighted multisensory integration during dynamic postural tasks being highly dependent on the direction of visual cues during tilt and less dependent on tonic SPH offsets. These techniques could be used to examine vestibular and visual interactions within clinical populations, particularly those with visual vertigo and dizziness.

## 1. Introduction

One of the first stages for the neural integration of visual sensory signals, elicited by moving scenes with respect to head motion and vestibular responses to head motion, occurs in the vestibular nuclei. The non-linear integration of visual and vestibular sensory signals appears to have common properties across species [1,2,3,4]. Thus, the non-linear weighting of visual sensory systems, when combined with vestibular signals in the vestibular nuclei, could profoundly influence the next stage of sensory integration, when visual and vestibular signals are integrated with neck proprioceptive signals [5], and then used to generate a motor response to body movements and the visual scene motion. A result of this interaction is that if a subject views a scene moving in the roll plane, the subject feels they have been tilted in the opposite roll direction and responds by tilting the trunk in the direction of roll stimulation [6,7], thereby down-weighting leg and trunk proprioceptive inputs indicating no tilt occurred. A similar response to visual motion occurs in the pitch plane [8]. Furthermore, when simultaneously pitched in the same direction by a support-surface pitch rotation, subjects overreacted as if the proprioceptive feedback gain was too low [8].

Haggerty et al. [9], Niehof et al. [10] and Anastasopoulos and Mergner [5] have suggested, based on modeling approaches, that visual, vestibular and proprioceptive inputs are combined using the same neural integration process. This neural process, prolonging a motor response, was first demonstrated for the vestibular system by examining the differences and similarities between cupular deviations based on the modeling of the semicircular canal and cupular dynamics [11] and response time constants of vestibular afferent fiber activity and nystagmus [12]. It was noted that a longer time constant was normally observed for nystagmus induced by whole-body rotations and that there was a shortening of the nystagmus time constant with reduced afferent activity following unilateral peripheral vestibular loss [13]. Although both Haggerty et al. [9] and Niehof et al. [10] assumed that the same type of neural integrator-shaped responses to a rotating visual scene (a circular vection (CV) stimulus [6]) and galvanic peripheral vestibular stimulation, there were important differences between their experimental protocols. The subjects of Haggerty et al. [9] were standing with their head unconstrained, and trunk tilt was measured as the primary response variable. In contrast, the subjects in the study of Niehof et al. [10] sat with their head fixed, and the subjective visual vertical (SVV) was the primary measured variable. These differences, specifically proprioceptive constraints, may account for the lack of a common neural integrator being observed by Niehof et al. [10]. Instead, in a simulation model, CV yielded a longer neural integrator time constant (7 s) compared to 4 s for galvanic stimulation.

In fact, the most common method used to measure the influence of CV on visual–vestibular perceptions is to seat subjects and ask them to adjust their SVV for different inclinations of their whole body turned out of vertical [14,15]. An increase in the mean perceived vertical offset of the SVV with respect to true vertical and its variance occurs with increasing angle of whole-body tilt. The increase in the variance of SVV estimates is correlated with the absence of ocular vestibular evoked myogenic potentials (o-Vemps) in bilateral vestibular deficit patients with utricular deficits [16]. It has proven difficult, however, to show changes in mean SVV estimates with the amount of unilateral vestibular deficit except under conditions of eccentric rotations when one utricle is at the center of rotation and effectively not stimulated [17,18].

Given these limitations of SVV in identifying unilateral peripheral vestibular loss, we developed a new test of the subjective postural horizontal (SPH) in which subjects had to return a 2° tilted platform to their SPH using a hand-held controller [19]. Based on the significant differences observed with this test between healthy normal subjects and those with complete unilateral loss due to vestibular neurectomy, we assumed that these differences would be enhanced with CV stimuli. As a first step in the current study, we measured head, trunk and pelvis angle changes similarly to the studies of Haggerty et al. [9], but an important difference in that we analyzed three upper body segments compared to one (head, trunk and pelvis) and probed the SPH at the same time. This enabled us to examine increasing upper body tilt responses when CV was present and to determine if a proprioception mismatch had occurred due to the ongoing CV. Such an SPH probe has the advantage that it does not require the display of the vertical bar of the SVV to be present, consequently interrupting the optic flow of the CV stimulus.

Thus, the purpose of the current study was to investigate the effect of dynamic visual signals elicited by CV on the phasic responses to roll tilt and on the steady state perception of support surface horizontal. We hypothesized that, firstly, CV would alter upper body segment responses occurring during support-surface tilt and, secondly, the platform steady state SPH position would also be altered in the direction of CV. Thirdly, we hypothesized that the effects of CV in the congruent directions on the body lean due to CV and platform tilt would be greater than the effects for the incongruent directions, and, fourthly, that the effects of CV on steady state body tilt induced by the platform movement would be less than the effects on the perceived SPH.

## 2. Methods

Ten young, healthy adults (mean age 21.8 years ± 2.6 (sd), 6 female) volunteered to participate in this prospective laboratory study, which was approved by the University of British Columbia Research Ethics Board, approval number H15-02398. All participants gave informed written consent to participate. They reported no known neurological or orthopedic disorders that may have affected balance nor any perceived dizziness, vertigo or motion sickness. The subjects all had normal hearing. The size of the study group, 10, was based on the number of subjects typically used for other studies with immersive visual environments, for example, Duh et al. [20].

**Figure 1:**
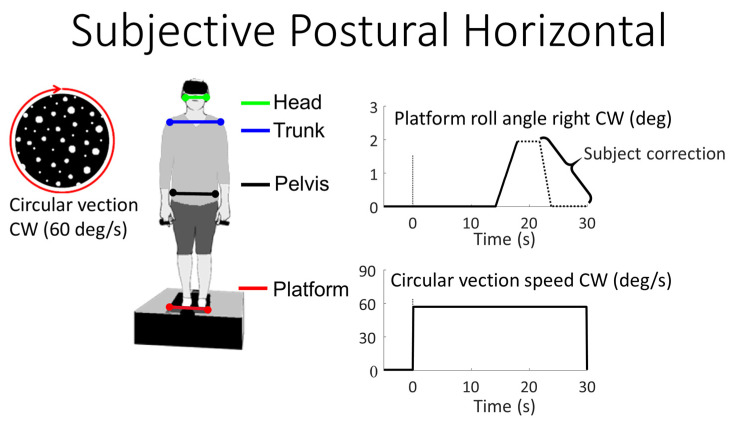
Schematic diagram of the experimental setup.

Participants stood barefoot on a custom-built rotating platform (Figure 1). Their feet were lightly strapped into heel guides to ensure their feet remained in the same position throughout the experiments. The stance width between the medial borders of the feet was always 14 cm. The roll axis of the platform was located equidistant between the feet at the height of the ankle joints. In some trials, the participants were required to reset the platform to their subjective postural horizontal (SPH). For this purpose, the platform was first rotated left (ccw) or right (cw) 2° at 0.6°/s., starting at 15 s into the recording (see Figure 1, right). Approximately 1 s after the rotation ceased at 3.3 s, a tone indicated that participants should rotate the platform back to their perceived horizontal using a hand-held controller, which changed platform roll position at 0.8°/s, see Figure 1, right. We chose this value of 0.8°/s in order to have approximately equal speeds for the initial rotation and subject correction (see Figure 1, right), as subjects required several correction segments to reach their SPH.

**Figure 2:**
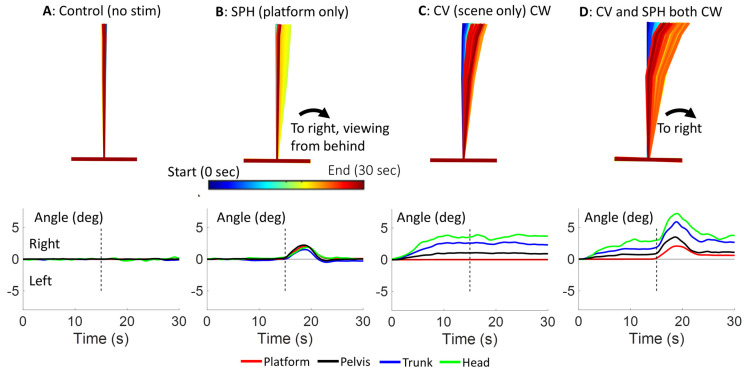
Illustration of the main 4 of the 9 experimental conditions (see Methods): (**A**) no stimulus condition; (**B**) the effect of right platform rotation alone and SPH is measured; (**C**) the effect of right CV alone; (**D**) the combined effect of congruent right CV and platform rotation with SPH being measured. The upper row of stick figures shows a typical subject response. The time coding for the stick figures is shown in column B. The lower row shows average population traces over time of roll angle movements of the platform, pelvis, trunk and head for the 4 conditions of the stick figure. For display purposes, only the traces have been filtered with a running average filter over 1 s, equal to the minimum averaging interval used to compare CV effects.

Visual stimuli were provided in a head-mounted display (Oculus Rift, Menlo Park, CA, USA). A custom-built random dot pattern (see Figure 1) was displayed to the participants (Worldviz, Santa Barbara, CA, USA). The pattern rotated either CW or CCW at 60°/s over the complete recording time (see Figure 1, lower right) to create a circular vection (CV) sensation [6,7]. The following 5 trial types were presented (see Figure 2):

1. Stationary dot pattern while standing on the platform (control condition) for 30 s.
2. CW or CCW CV random dot pattern rotating in the roll plane at 60°/s for 30 s.
3. CW or CCW SPH test starting 15 s after recording start. Trial duration 30 s. Stationary visual scene projected.
4. Congruent CV to SPH test direction. That is, CV and SPH test rotation are both in the same CW or CCW direction. Trial duration 30 s. SPH test rotation starts at 15 s.
5. Incongruent CV to SPH test direction. That is, CV and SPH test rotation both rotate in opposite CW and CCW directions. Trial duration 30 s. SPH test rotation starts at 15 s.

Items 2–5 of these trial types represent 8 conditions. Each participant received these trial types in a pseudo-random order that was repeated a minimum of 4 times for each participant.

Roll angular displacement of the head, trunk, pelvis and platform was calculated from 3D data obtained by a motion analysis system sampling light-emitting body markers at 100 Hz (Optotrak, Waterloo, ON, Canada). The markers were placed bilaterally on the orbital bones of the head, the acromion processes of the trunk, the anterior aspects of the pelvic iliac crest, and the platform (see Figure 1). The angles were bias-corrected in the roll plane based on the value at the beginning of each recording. The platform roll drive voltage, the platform roll angle potentiometer, and the participants’ drive signal for SPH were sampled at 2 kHz with Spike2™ software (Cambridge Electronic Design, Cambridge, England).

**Figure 3:**
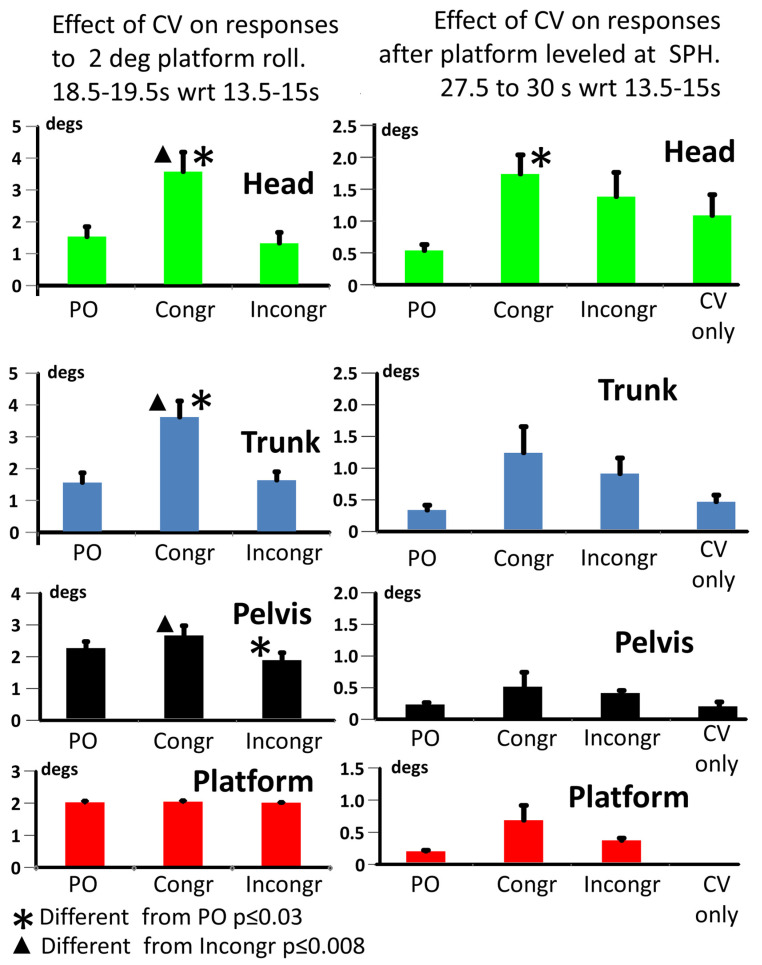
Population average responses to platform tilt (on the **left**) and measured SPH (on the **right**). The mean amplitudes of the measurement are represented by the height of the columns and the standard error of the mean (sem) by vertical bars on the columns. On the left, the effect of congruent and incongruent CV on body tilt is shown as the change in measurements between the mean value over 13.5–15 s (before platform movement started) compared to the mean value over 18.5–19.5 s (after platform movement ceased at 18.3 s). As shown in the lower part of Figure 2, the response to CV does not change between these time intervals. On the right, the effect of congruent and incongruent CV on the subjects’ set SPH and body lean adopted at 27.5 to 30 s after trial onset is compared to values when no CV is present (PO) and when no SPH test was performed (CV only). Note that the axis sensitivity is greater on the right.

**Figure 4:**
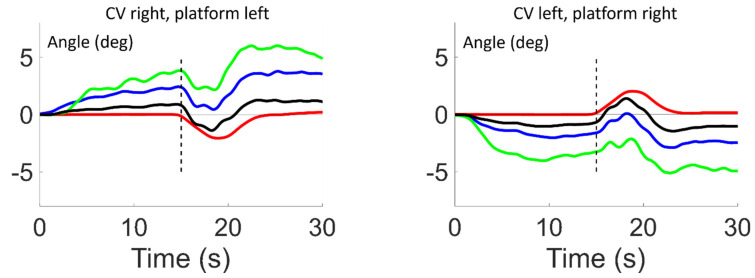
Average population time traces of platform, pelvis, trunk and head roll angle for the incongruent conditions of SPH and CV. On the left, when CV is CW and SPH platform tilt is CCW, on the right, when CV is CCW and SPH platform tilt is CW. The traces have been filtered by a 1 s running average filter. The color coding of the traces is the same as that in Figure 2.

Averages of the following measurement variables, head, trunk, pelvis and platform angle, were obtained over the following time intervals:

1. Pre-tilt: 13.5 to 15 s after recording start. That is, just before the SPH test started. This value was used to determine the amount of body lean induced by CV prior to tilt.
2. Post-tilt: 18.5 to 19.4 s after recording start, that is, 3.5 to 4.4 s after the start of the SPH platform rotation, which terminated after 3.3 s (at 18.3 s). This value was referenced to values pre-tilt and used to determine the change in body lean induced by the platform movement when CV was present. This interval (18.5 to 19.4 s) included the interval for which body segments had maximum displacements (see Figure 2, Figure 3 and Figure 4).
3. Post-SPH: 27.5 to 30 s after each trial started. Measures at this time-point were referenced to those of pre-tilt and used to quantify the effect of CV on the SPH. Measures at this time point were also compared with the body lean measures for CV alone.

Pre-tilt, post-tilt, and post-SPH measures were averaged for each participant across trials within each condition and then rectified to allow for maximum comparisons between conditions. If marker data were missing for more than 0.5 s sequentially in any of the three measurement windows, the trial was removed from further analysis. In addition, the first trial of each condition was removed from all analyses to reduce first trial effects [21]. A 2 direction (cw and ccw) by 3 condition (SPH alone, congruent, incongruent) repeated measures ANOVA was used to assess the effect of CV in response to platform tilt after checking that these measures were normally distributed. If sphericity was violated, Greenhouse–Geisser corrections were applied. Post-hoc parametric analyses were used to determine the significance of differences between the conditions, and a Bonferroni correction was applied to account for multiple comparisons. Post hoc comparisons focused on the main effects of condition. Significance was set at *p* ≤ 0.05 after the Bonferroni correction.

## 3. Results

Figure 2 provides an overview of the results for the condition when CV and the platform tilt for SPH are both congruent and clockwise. The SPH response for the condition named “platform only” (PO) was characterized by the body leaning in the direction of the platform inclination. The body lean was then corrected to near vertical as subjects returned the platform to their perceived horizontal. As shown by the “CV only” kinematic figure and average population traces of Figure 2, body lean in the direction of CV was greatest for the head, then the trunk, and least for the pelvis. The lean increased over the first 10 s and then remained relatively constant so that there was no statistical difference between body lean at 13.5–15 s and that at 27.5–30 s (1.08, 0.44 and 0.18°, for the head, trunk and pelvis respectively; see column 5 Table 1C).

### 3.1. Effects of CV on Upper Body Responses to Platform Tilt

Two main effects were observed when CV and SPH were combined in the same (congruent) direction. Firstly, there was an increase in trunk and head lean compared to that induced by the PO (Figure 3, Table 1B columns 2 and 3) and CV (Figure 3, Table 1B columns 3 and 5) conditions alone. This change in body lean was not statistically significant for the pelvis (Table 1B column 7). Secondly, body lean decreased as subjects returned the platform to their SPH, therefore moving with the platform (Figure 2). At the time subjects set their SPH, the amount of body lean was greater for the congruent condition than for the PO condition; however, this difference was only significant for the head (Figure 3; Table 1C columns 3 and 7).

Figure 4 shows the effect of combining CV and SPH conditions in opposite directions (incongruent). The differences in the responses to the tilt in Figure 2 and Figure 4, as well as the calculated differences in Figure 3 and Table 1B, all show that the magnitude of the responses to tilt in terms of changes in head, trunk and pelvis roll angles were far greater for the congruent than the incongruent conditions (Figure 4, and Table 1B columns 3 and 4). In contrast to the congruent condition, the pelvis roll lean was less for the incongruent condition than the lean induced by the PO condition (and therefore the congruent condition as well; Figure 3 left and Table 1B columns 3 and 4).

In summary, CV induces an increased head and trunk lean in response to the 2° platform movement when platform rotation is congruent with CV. However, when the platform rotation is incongruent to the CV, the head, trunk and pelvis rotate less than for the congruent condition. For the pelvis, the rotation is less than that for the PO condition too, implying that pelvis rotation is in the opposite direction to that of the platform.

### 3.2. Effects of CV on SPH Responses

Figure 2 and Figure 4 show the subjects’ movement time course and end position when returning the platform to their perceived horizontal (SPH) under congruent and incongruent conditions. The associated values at 27.5–30 s, representing the end of the SPH task, are illustrated in Figure 3 right and presented in Table 1C. There were minor differences in set SPH platform position values across congruent and incongruent conditions compared to the platform only (PO) condition, with the largest differences observed for the congruent condition (see Figure 3 right, but there was no main effect of condition observed, *p* = 0.116). In contrast, the head lean angle was significantly different for the congruent conditions (*p* = 0.012) and not significantly different for the incongruent condition (*p* = 0.075), compared to the PO condition (Figure 3 right and Table 1C). However, these body lean magnitudes at 27.5–30 s are similar to those of CV alone. Furthermore, there was no difference between the body lean magnitudes for the congruent and incongruent conditions (Table 1C).

## 4. Discussion

The current study investigated the effect of dynamic visual stimuli elicited by CV on the phasic responses to support-surface roll rotations and on the ensuing perception of support surface horizontal in the frontal plane (roll). The latter balance assessment was originally developed as a test for utricular responses [19]. Roll CV had a significant effect on the amplitudes of head and trunk responses to lateral platform tilt but a weaker effect on the SPH of the support surface. Response amplitudes during tilt indicate enhanced reactions for congruent and reduced reactions for incongruent CV, both of which were different from responses to CV alone and platform tilt (PO) alone.

### 4.1. Effects of CV on Phasic Body Position and Platform SPH Position

Roll plane CV stimuli significantly influenced upper body tilt responses in a “top-down” mode. That is, the size of absolute and relative response amplitudes was greatest for the head and least for the pelvis. Interestingly, the rotation at the pelvis, once the platform SPH had been set, was similar in amplitude to that of the platform (Figure 3, right, and Table 1C, rows 4 and 5), suggesting that the lower body position was set in a “bottom-up” mode with proprioceptive reflexes dominating lower body position.

To date, most work on postural responses altered by moving visual scenes has involved linearly moving scenes. For example, the classic paper of Nashner and Berthoz [22] described the enhancement of leg postural responses of standing subjects to linear motion of the surroundings. However, more recently, studies [8] have examined the effect of increasing velocity of backward pitch rotation of the visual surroundings on responses to backward pitch rotation of the support surface. The increased effect on body centre-of-mass displacement in the Wang et al. [8] study and in our studies on upper body motion suggests that there might be common pathways controlling vestibular spinal corrections to support surface displacements and visual influences on postural responses elicited by an illusion of self-motion. These common pathways could well originate in the vestibular nuclei where neurons respond to both the motion of the visual surround and body acceleration in the same plane [1,2]. The question arises whether the proposed linear interaction between visual and vestibular signals [3] still applies when the surround motion is considerably greater than body rotation as in the current study (60 compared with 0.6 deg/s). With visual field velocities as low as 20 deg/s, first-order vestibular nuclei neurons can be driven into saturation [1].

In contrast to the effect on upper body lean, there was no statistical difference between the SPH values set when CV was present or not (0.65 vs. 0.17 deg). Previous work has shown that vision (eyes open versus closed) has no major influence on the perception of the horizontal under these test conditions [19]. Therefore, it is not surprising that little influence was seen with CV. Another reason for the lack of influence of CV on lower leg and pelvis rotation could be that the proprioceptively triggered responses in the legs are increasingly delayed at the ankle, knee, hip and neck, respectively [23], making it more difficult to coordinate joint stretch responses with visual signals. This may be the reason that visual modulation of leg muscle responses to support surface tilt commences 350 ms after roll tilt onset [24]. Another factor that needs to be taken into account when considering the influence of CV on head tilt is that neck stretch reflex responses can be suppressed [25], leading to increased head motion with support surface rotation. This may enable a better measurement of head tilt using utricle responses. Such a neck proprioception gain modification process would be useful for restoring graviception during the compensation for vestibular loss [26].

Based on our results, the effect of CV on the amplitudes of postural responses to platform tilt, occurring prior to when the subject was required to correct platform position to their SPH, was significantly greater than the actual corrected platform tilt. When the increase in body lean was referenced to the CV only condition and compared with that of the response to the platform tilt, no difference was observed for the incongruent condition. For the congruent condition, the difference for head, trunk and pelvis was significantly larger and further supported our conclusion that congruent CV and support surface tilt have additive effects on perceived body tilt.

### 4.2. Congruent vs. Incongruent Directions

Perceived platform horizontal, and to a far greater extent, head tilt, are influenced by CV, with the largest effects on perceived horizontal, contrary to our original hypothesis, being observed during congruent visual and platform rotation. During the congruent conditions, the platform tilt further rotated all aspects of the body in the direction of the CV. However, for the incongruent condition, there appears to be a bimodal movement, where the lower body is affected by the platform tilt enough to overcome the lean caused by the CV. Further work is needed to examine this effect, specifically examining the interaction between support surface tilt and direction and velocity of dynamic visual stimuli, and more generally, if a similar effect to that we observed for roll is observed for the pitch and yaw axes. In addition, because of the interaction between vestibular and visual signals in the vestibular nuclei, which we hypothesize forms the basis of the enhanced influence of CV on support surface tilt responses, the question arises whether these techniques hold the promise of being useful to examine central vestibular disorders in persons with brainstem lesions.

### 4.3. Study Limitations

We have concentrated on comparing our results to the interaction between visual and vestibular signals occurring in the CNS, specifically to the pattern of interactions occurring in the vestibular nuclei. Clearly, there are other sites in the brain where such interactions between vestibular and visual signals occur, for example, in the vestibular cortex [27]. Another limitation of this study is the size of the study population (10 subjects). The post-hoc analyses generally led to significant differences for the head and trunk movement amplitudes between each condition but not for the smaller amplitudes of the pelvis. We assume the results for the pelvis would become significant with a greater number of participants.

### 4.4. Conclusions

Roll plane CV stimuli significantly influence the roll orientation of upper body segments during responses to support surface roll tilt. The perceived support surface horizontal position is altered to a lesser extent. These results support the hypothesis of weighted multisensory integration during upright stance. In addition, subjective postural horizontal assessments and continuous visual perturbations may be useful to examine deficits in visual–vestibular interactions within clinical populations, particularly those with visual vertigo, and lead to a more specific test of otolith function than subjective visual vertical [28].

## Figures and Tables

**Table 1:** Angle displacements of platform and body segments (mean ± sem) at different measurement times for the 4 stimulus conditions. Note that the RM ANOVA was only run on the first 3 conditions (PO, Congruent, and Incongruent). Platform and body angles averaged over (A) 13.5 to 15 s after CV stimulus onset and prior to SPH platform movement (pre-tilt); (B) the difference between post-tilt (18.5 to 19.5 s) and pre-tilt; and (C) the difference between post-SPH (at 27.5 to 30 s) and pre-tilt. Column 6 in sub-tables A, B and C lists the results from the RM ANOVA (main effect of condition only), probability (*p*) and F values for the significance of the main effects (2, 16 degrees of freedom, unless corrected by the Greenhouse–Geisser correction). Columns 7 to 9 lists the *p* values for the Bonferroni corrected post-hoc comparisons. Cells highlighted in grey indicate a non-significant main effect of condition. The CV only values in column 6 document the influence of CV on average body angles at the 3 measurement time points.

**A** **Time** **13.5 to 15 s**	**Platform Only**	**Congruent Platform** **and CV**	**Incongruent Platform and CV**	**CV** **Only**	**Main Effect of Condition**	**PO vs. Con**	**PO vs. InCon**	**Con vs. InCon**
Head	0.45 ±0.97	3.65 ±0.73	3.08 ±0.75	3.42 ±0.55	**F = 11.411,** ***p* = 0.003**	**0.008**	**0.047**	0.807
Trunk	0.32 ±0.06	1.67 ±0.32	1.71 ±0.26	1.81 ±0.32	**F = 15.871,** ***p* = 0.001**	**0.017**	**0.003**	1
Pelvis	0.15 ±0.05	0.78 ±0.23	0.68 ±0.17	0.76 ±0.15	**F = 6.987,** ***p* = 0.028**	0.096	0.066	0.528
Platform	0.03 ±0.01	0.03 ±0.01	0.02 ±0.00	0.01 ±0.00	F = 2.938, *p* = 0.107	1	0.776	0.013
**B** **Time** **18.5 to 19.5 s cf 13.5 to 15 s**	**Platform Only**	**Congruent Platform and CV**	**Incongruent Platform and CV**	**CV** **Only**	**Main Effect of Condition**	**PO vs. Con**	**PO vs. InCon**	**Con vs. InCon**
Head	1.51 ±0.31	3.56 ±0.62	1.30 ±0.35	0.64 ±0.17	**F = 16.677,** ***p* = 0.001**	**0.021**	1	**<0.001**
Trunk	1.53 ±0.30	3.57 ±0.51	1.60 ±0.27	0.25 ±0.06	**F = 14.262,** ***p* = 0.004**	**0.028**	1	**0.002**
Pelvis	2.23 ±0.21	2.63 ±0.31	1.85 ±0.24	0.14 ±0.05	**F = 8.952,** ***p* = 0.009**	0.381	**0.030**	**0.008**
Platform	2.02 ±0.01	2.04 ±0.01	2.03 ±0.01	0.00 ±0.00	F = 1.981, *p* = 0.192	0.355	1	0.096
**C** **Time** **27.5 to 30 s** **cf 13.5 to 15 s**	**Platform Only**	**Congruent Platform and CV**	**Incongruent Platform and CV**	**CV** **Only**	**Main Effect of Condition**	**PO vs. Con**	**PO vs. InCon**	**Con vs. InCo**
Head	0.52 ±0.10	1.74 ±0.31	1.38 ±0.39	1.08 ±0.33	**F = 6.045,** ***p* = 0.011**	**0.012**	0.075	1
Trunk	0.31 ±0.08	1.22 ±0.42	0.89 ±0.25	0.44 ±0.11	F = 2.328, *p* = 0.161	0.239	0.034	1
Pelvis	0.21 ±0.03	0.49 ±0.23	0.39 ±0.04	0.18 ±0.07	F = 1.161, *p* = 0.314	0.760	0.002	1
Platform	0.17 ±0.02	0.65 ±0.23	0.34 ±0.04	0.00 ±0.00	F = 3.068, *p* = 0.116	0.234	0.011	0.683

## Data Availability

If requested, analysis data will be made available.
